# Protective Mechanism of Proprotein Convertase Subtilisin-Like Kexin Type 9 Inhibitor on Rats with Middle Cerebral Artery Occlusion-Induced Cerebral Ischemic Infarction

**DOI:** 10.1155/2022/4964262

**Published:** 2022-03-26

**Authors:** Shengxiong Pu, Chaojun Jia, Zhimin Li, Yunhua Zang

**Affiliations:** ^1^Department of Neurology, Affiliated Hospital of North Sichuan Medical College, Nanchong, China; ^2^Qingdao Hospital of Traditional Chinese Medicine (Qingdao Hiser Hospital), Qingdao, China

## Abstract

The aim of this study was to research the mechanism of proprotein convertase subtilisin-like kexin type 9 (PCSK9) inhibitor in neural protective effect on rat cerebral ischemic reperfusion injury (I/RI). The transient middle cerebral artery occlusion (tMCAO) model of rats was prepared by the suture method, and PCSK9 inhibitor was injected intraperitoneally immediately after I/R. The rats were scored for neurological deficits and the cerebral infarction volume was measured. The brain tissues were collected and western blot (WB) was used to detect the expression of PCSK9. The rat cortical neural stem cells were treated with oxygen glucose deprivation (OGD) to establish a cell model of ischemia/reperfusion. WB was used to detect the expression of PCSK9 and the apoptosis-related pathway proteins. After interfering with the expression of PCSK9 siRNA, the cell viability (cell counting kit-8 assay) and apoptosis (TUNEL staining, Annexin V/PI method) were detected, and the cell proliferation was detected by EdU staining and flow cytometry. The expression of PCSK9 in the brain tissue of the MCAO group was dramatically increased. PCSK9 inhibitor can improve neurobehavioral scores and reduce apoptosis and infarct volume. An OGD model of neural stem cells in vitro was constructed. Inhibiting PCSK9 with si-PCSK9 can increase cell viability, promote cell proliferation, and also reduce cell apoptosis. Inhibition of PCSK9 can decrease the cerebral infarct volume in rats with cerebral I/RI and improve the neural function. Mechanically, inhibition of PCSK9 can lead to the decrease of nerve cell apoptosis and promotion of cell proliferation.

## 1. Introduction

The current population aging has become a global problem. Correspondingly, ischemic stroke, as one of the main cerebrovascular diseases that threaten human health, has the characteristics of high morbidity, high recurrence, high disability, and high mortality [1]. Because of the existence of collateral circulation in the ischemic penumbra, it is not completely infarcted, and neuronal function is still possible to recover. It has always been a hot spot in the study of acute ischemic stroke. The early restoration of blood flow is of great significance for reducing the infarct size, restoring patient dysfunction, and reducing mortality and disability [2]. After the blood supply of the tissues and organs is interrupted and blood flow is restored, the functions of the tissues and organs are not restored or even worsened, and the dysfunction and structural damage are more serious, which is called ischemic reperfusion injury (I/RI) [3]. The mechanism of I/RI is very complicated. It is currently believed that various mechanisms such as inflammation, apoptosis, necrosis, oxidative stress, calcium overload, and free radical generation are involved [4]. Thrombolysis is a clinically effective treatment method, but its limited time window limits its clinical application, making the development of new and effective targets and drugs for the treatment of ischemic stroke particularly important.

Proprotein convertase subtilisin-like/kexin type 9 (PCSK9) was discovered by Seidah et al. [5] in 2003, and it is the ninth molecule in the proprotein convertase (PC) family. PCSK9 is mainly produced by the liver and then released into the blood. It returns to the liver through the circulation and binds to the low-density lipoprotein receptor (LDLR) on the surface of liver cells to form a LDLR-PCSK9 complex, which is digested by hepatocytes [6], leading to a decrease in LDL clearance and an increase in LDL-C in the blood. PCSK9 is mainly expressed in the kidney, small intestine, liver, and other organs. PCSK9 is also expressed in the cortex and hippocampus [7]. A large number of literature studies have pointed out that the mechanism of PCSK9 in the nervous system is different from that of the liver. It is involved in such as inflammation and oxidative stress [8]. In addition, foreign scholars believe that PCSK9 as a neuro apoptosis regulating converting enzyme is also involved in mechanisms such as apoptosis [8, 9].

Pep2-8 is a PCSK9 inhibitor, which can selectively bind to PCSK9 and block various biological effects of PCSK9 [10]. Therefore, we hypothesized that inhibition of PCSK9 expression after I/R may rescue neurons away from ischemia-induced apoptosis via downregulating caspase-3 pathway. In our study, we established a tMCAO model in SD rats to observe the interventional effects of PCSK9 inhibitor on neurobehavioral scores and cerebral infarct volume after ischemia/reperfusion in SD rats. A neural stem cell OGD in vitro was constructed to explore the effects and potential mechanism of PCSK9 on cell proliferation and apoptosis.

## 2. Methods and Materials

### 2.1. Experiment Animal

A total of 30 adult male Sprague-Dawley (SD) rats (body weight 250–280g) and fetal rats at 18 days of gestation were provided by the Experimental Animal Center of North Sichuan Medical College, and adult male SD rats were used for in vivo animal experiments. Fetal SD rats are used for the extraction and culture of neural stem cells in vitro. This study was approved by the Animal Ethics Committee of North Sichuan Medical College Animal Center.

### 2.2. Animal Grouping and MCAO Model

SD rats were randomly divided into the following: (1) sham group: rats underwent sham operation. (2) MCAO group: SD rats were reperfused after 2 hours of ischemia. (3) PCSK9i (Pep2-8) treatment group: reperfusion after 2 hours of ischemia, and intraperitoneal injection of 10 *μ*g/kg PCSK9 inhibitor immediately after reperfusion. The operation method refers to the MCAO method: (1) after the rats were weighed, they were anesthetized by intraperitoneal injection with 4% sodium pentobarbital. (2) The anesthetized rat was fixed on the operating table in a supine position. After the neck was shaved and disinfected, an incision with a length of about 2 cm was cut in the middle. (3) The blood vessels and the vagus nerve were separated, and the left common carotid artery, external carotid artery, and internal carotid artery were exposed. (4) The proximal end of the external carotid artery and the common carotid artery were ligated, a single knot was tied at the distal end of the common carotid artery, and the internal carotid artery was clamped with an arterial clip. (5) A small gap was cut midway above the ligation of the proximal end of the common carotid artery, and the prepared nylon suture was slowly advanced through the incision to the internal carotid artery. At the same time, the arterial clamp on the internal carotid artery was loosened. (6) When the length of the thread plug was 18 mm–20 mm, the resistance can be obviously felt. At this time, the opening of the middle cerebral artery was blocked and the thread plug was fixed. (7) After blocking for 2 hours, the single knot was loosened, the nylon thread plug was slowly pulled out, and the distal end of the common carotid artery was ligated, finally restoring blood circulation and achieving reperfusion. (8) After suturing the skin, debridement and disinfection were performed. Sham group: arterial embolization by inserting a thread plug was not performed, and the other operations were the same as those in the MCAO group.

### 2.3. Modified Neurological Severity Scores

mNSS is one of the most commonly used scales for evaluating neurological function after stroke, including four categories: motor, sensory, reflex, and balance. The rat was assigned a value when it cannot complete the action or meets the item. The greater the score, the more severe the damage. The scoring system referred to the method in the previous literature [11].

### 2.4. Rotarod Test

The Rotarod test was mainly used to evaluate the coordination and balance of rodents. The rat was placed on a roller, the speed was gradually increased from 4 rpm to 40 rpm within 5 minutes, and the incubation period was recorded from the beginning to the time of the rat falling. The rats were trained every day for three days before modeling, and the average was measured three times.

### 2.5. Frozen Section of Mouse Brain

On the 7th day after modeling, the rats were anesthetized with 4% sodium pentobarbital. After opening the chest, the right atrial appendage was cut and slowly 50 ml of normal saline was infused through the left ventricle until the right atrial appendage was clear. Then, the brain tissue was taken out and quickly put in isopentane precooled in the ultralow temperature refrigerator for about 1-2 minutes. Then, the rat brain was taken out, marked, and put in the ultralow temperature refrigerator for later use. When slicing, the rat brain was taken out and placed in a microtome for about 1 hour. After adaptation, the slice was embedded in OCT (Keygen, Nanjing, China) with a thickness of 20 *μ*m.

### 2.6. TTC Staining

The brain tissue sections were incubated with 2% TTC solution (Jian Cheng, Nanjing, China) at 37°C for 20 minutes, and then the excess TTC regent was drained. The brain slices were fixed with 4% paraformaldehyde.The Image J analysis system was used to quantify the infarct volume.

### 2.7. Western Blot

After the brain tissue or neural stem cells total protein was extracted and determined, sodium dodecyl sulphate-polyacrylamide gel electrophoresis (SDS-PAGE) and membrane transfer were performed. After blocking, the primary antibody (PCSK9, Abcam, Cambridge, MA, USA, mouse, 1 : 1000; Bax, Abcam, rabbit, 1 : 1000; Bcl-2, Abcam, rabbit, 1 : 1000; caspase-3, Abcam, rabbit, 1 : 1000; and *β*-actin, Abcam, 1 : 5000) was added and incubated overnight at 4 °C. The secondary antibody conjugated with horseradish peroxidase was incubated for 1 hour at room temperature, and the membrane was washed 3 times with tris buffered saline-tween (TBST). Then,the membrane was incubated with electrochemiluminescence (ECL) solution (Camilo, Nanjing, China) for 1 minute and exposed for 5–20 minutes in the cassette for developing and fixing. AlphaEaseFC grayscale analysis software was used to analyze the band grayscale scanning, and the results were expressed as the target protein by the gray value of the sample band/corresponding internal reference gray value. In this study, *β*-actin was used as an internal reference.

### 2.8. Isolation of Primary Cortical Neural Stem Cells

Primary neural stem cells were taken from the cerebral cortex of rat embryos at 18 days of pregnancy. The cerebral cortex was digested with 0.25% trypsin (Camilo, Nanjing, China), and then the cell suspension was inoculated into a 6-well plate under 95% air and 5% carbon dioxide conditions. The cell culture medium was DMEM containing 10% fetal bovine serum (FBS) and cytosine-D-arabinofuranoside (10 *μ*M). In OGD treatment, cortical neurons were cultured in Dulbecco's modification of Eagle's medium (DMEM, Thermo Fisher Scientific, Waltham, MA, USA) for 12 hours under normal conditions and then incubated for 2 hours under hypoxic conditions (95% N_2_ and 5% CO_2_) with 0.5 mmol/L of glucose and sodium dithionite, and the medium was changed every two days and cultured for 7 days.

### 2.9. Transfection

The neural stem cells in the logarithmic growth phase were seeded in a 96-well plate to grow the cell density to 60%∼80%, and the cells were transfected according to the Lipofectamin^2000^ (Keygen, Nanjing, China) instructions. siRNA-PCSK9 and negative control were diluted with 250 *μ*L serum-free medium and incubated at room temperature for 5 minutes. Lipofectamin^2000^ 5 *μ*L was diluted with 250 *μ*L opti-MEM without serum and incubated at room temperature for 5 minutes. Then, the above liquid was mixed, incubated at room temperature for 20 minutes, and then added to the cell culture well. Cells were incubated in a 37 °C, 5% CO_2_ incubator for 6–8 hours. After culturing for 24–48 hours, subsequent experiments were performed.

### 2.10. Cell Counting Kit-8 (CCK-8) Assay

The treated neural stem cells (2 × 10^4^ cells/100 *μ*L) were inoculated for 96 hours and 10 *μ*L of CCK-8 solution (Ye Sen, Shanghai, China) was added to each sample, 3 replicate wells were repeated. After incubating for 2–4 hours, the absorbance at 450 nm was measured with a microplate reader (BD-Biosciences Franklin Lakes, NJ, USA).

### 2.11. Cell Cycle Assay

The treated neural stem cells were cultured in a 6-well culture plate at 37 °C until the cells reached 80% confluence. The cells were collected with 70% precooled ethanol. After washing with phosphate buffered saline (PBS) twice, the cells were resuspended with 0.5 mL PBS and added with PI (Thermo Fisher Scientific, Waltham, MA, USA) for 30 minutes. Finally, the cells were analyzed by flow cytometry (BD-Biosciences, USA).

### 2.12. Flow Cytometry

The processed neural stem cells were collected first, and then the cells were suspended in a buffer containing 60 *μ*g/ml PI and Annexin V (Ye Sen, Shanghai, China) and kept at room temperature in the dark for 15 minutes.Subsequently, cell apoptosis was detected by ACS-Caliber flow cytometer.

### 2.13. TUNEL Staining

After the sections or cell slides were incubated with 2% hydrogen peroxide for 30 minutes, the cell membrane was permeated with Trixon X-100 (Keygen, Nanjing, China) for 30 minutes. Then, the TdT enzyme buffer (Keygen, Nanjing, China) was used to incubate at room temperature for 15 minutes, and then 50 *μ*L of TdT enzyme reaction solution was added dropwise and incubated at room temperature for 60 minutes. After washing with PBS, the reaction was terminated. Next, the peroxidase-labeled antibody was used for reaction at room temperature for 30 minutes. After 10 minutes of methyl green counterstaining, 4 fields of view were randomly selected with an image analysis system to count the number of positive apoptotic cells.

### 2.14. 5-Ethynyl-2′-Deoxyuridine (EdU) Staining

Neural stem cells treated with OGD were seeded in a 6-well plate at 1 × 10^5^ cells per well. After culturing overnight, the original medium was replaced with a fresh medium containing EdU (100 *μ*M, Camilo, Nanjing, China) for 2 hours, then the cells were fixed with acetone for 20 minutes. The Apollo reaction buffer with FITC fluorescein was incubated for 1 hour and then photographed with a laser confocal fluorescence microscope (Leica, Wetzlar, Germany).

### 2.15. Statistical Analysis

All data were expressed as the mean ± standard deviation ($\bar{X}$ ± SD) of three repeated experiments and analyzed by Statistical Product and Service Solutions (SPSS) 20.0 statistical software (IBM, Armonk, NY, USA). Student's *t*-test was used for comparison between the two groups. One-or two-way analysis of variance (ANOVA) was used for data analysis among multiple groups. The statistical analysis of behavioral data adopts repeated measure ANOVA. *p* < 0.05 is regarded as a statistically significant difference.

## 3. Results

### 3.1. Expression of PCSK9 Increases in MCAO Rats' Brain Tissue

**Figure 1:**
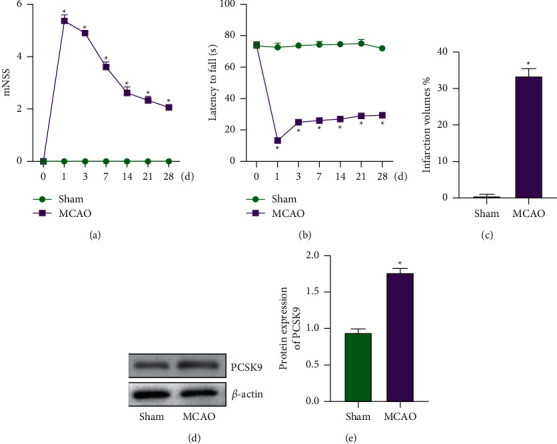
The expression of PCSK9 increases in MCAO rats' brain tissue. (a) Modified Neurological Severity Scores (mNSS). (b) Rotarod test. (c) TTC staining detected the infarct volume of brain tissue. (d, e) WB detected the protein of PCSK9 in brain tissue. ^*∗*^*vs.* sham group <0.05.

In order to verify the success of MCAO model, we performed neurobehavioral scores on the rats after modeling. It was found that the mNSS score of the rats increased in the MCAO group rats (Figure 1(a)), and the incubation period of the rat Rotarod test decreased (Figure 1(b)). On the 7th day of I/R, the brains of the two groups (sham/MCAO) were taken for TTC staining. It was found that the cerebral infarct volume of the rats in the MCAO group increased dramatically than the sham group (Figure 1(c)). The above results show that MCAO model was successful. Next, we used WB method to detect the expression of PCSK9 in the subventricular zone of rats. Compared with the sham group, the expression of PCSK9 in the MCAO group was dramatically increased (Figures 1(d) and 1(e)).

### 3.2. Protective Effect of PCSK9 Inhibitor on MCAO Rats

**Figure 2:**
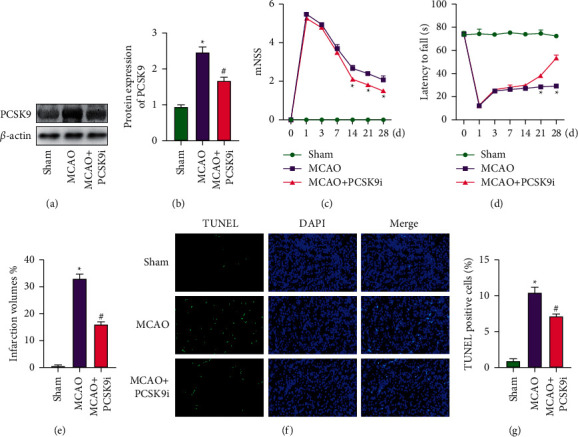
Protective effect of PCSK9 inhibitor on MCAO rats. (a, b) WB detected the protein of PCSK9 in brain tissue. (c) Modified Neurological Severity Scores (mNSS). (d) Rotarod test. (e) TTC staining detected the infarct volume of brain tissue. (f, g) TUNEL staining detected the apoptosis cell of brain tissue. ^*∗*^*vs.* sham group <0.05; # *vs.* MCAO group <0.05.

In order to explore the effect of PCSK9 on MCAO rats, we injected PCSK9 inhibitor into the intraperitoneal cavity. WB results showed that PCSK9 inhibitor can dramatically inhibit the expression of PCSK9 in brain tissue (Figures 2(a) and 2(b)). At the same time, we performed neurobehavioral scores on rats and found that PCSK9 inhibitor can reduce the score of mNSS and increase the incubation period of the Rotarod test (Figures 2(c) and 2(d)).TTC results showed that compared with the MCAO group, the infarct volume in the MCAO + PCSK9i group was dramatically reduced (Figure 2(e)). Next, we used TUNEL staining to detect apoptotic cells in rat brain tissue and found that compared with MCAO group, the percentage of apoptotic cells in MCAO + PCSK9i group decreased (Figures 2(f) and 2(g)). The above results indicated that PCSK9i can improve the neurological function of MCAO rats and reduce the volume of cerebral infarction and cell apoptosis.

### 3.3. Inhibition of PCSK9 Partially Inhibits OGD-Induced Apoptosis via Bax/Bc-l2 Signaling In Vitro

**Figure 3:**
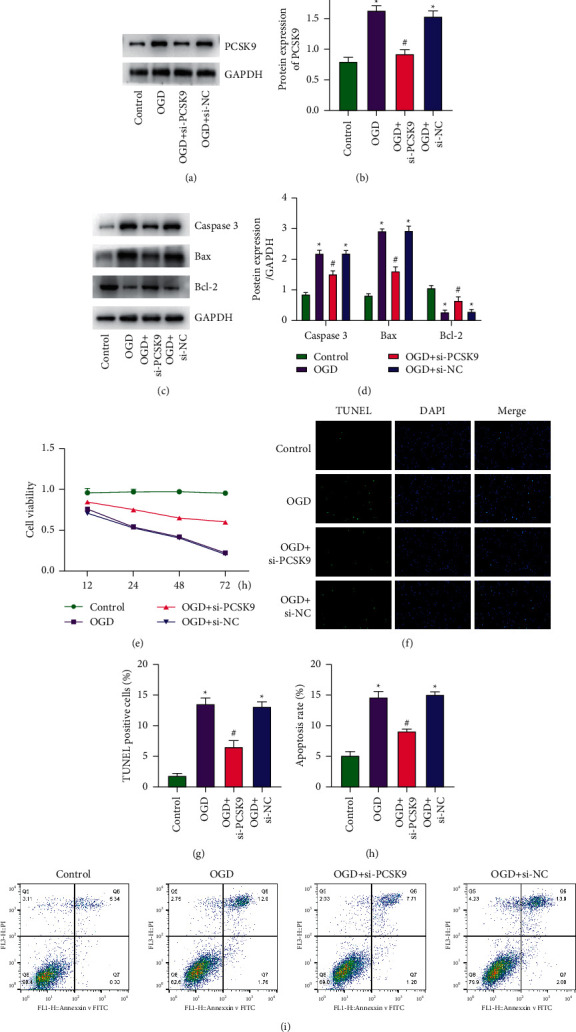
Inhibition of PCSK9 partially inhibits OGD-induced apoptosis in vitro. (a, b) WB detected the protein of PCSK9 in neural stem cells. (c, d) WB detected the protein of Bax, Bcl-2, and caspase-3 in neural stem cells. (e) CCK-8 assay detected the cell viability. (f, g) TUNEL staining detected the apoptosis of neural stem cells. (h, i) Flow cytometry detected the apoptosis rate of neural stem cells. ^*∗*^*vs.* control group <0.05; # *vs.* OGD + si-NC group <0.05.

We used 18-day-old fetal rat cortical neural stem cells to establish an in vitro cell model of ischemia and hypoxia. To explore the effect of PCSK9 on OGD-induced in vitro ischemic-hypoxic cell models, siRNA was used to knock down PCSK9. WB was used to detect the efficiency of knockdown, the results showed that the expression of PCSK9 in the OGD + si-PCSK9 group was dramatically reduced compared with OGD + si-NC group (Figures 3(a) and 3(b)). Furthermore, the apoptosis-related pathway Bax/Bcl-2 signaling was determined by WB assay, showing that OGD treatment significantly increased the expression of Bax and caspase-3 and decreased the Bcl-2 expression, but inhibition of PCSK9 markedly reduced the ratio of Bax/Bcl-2 and the level of caspase-3 (Figures 3(c) and 3(d)). CCK-8 assay was used to detect cell viability and it was found that compared with the OGD group, the cell viability of the OGD + si-PCSK9 group was improved (Figure 3(e)). The result of TUNEL staining showed that the ratio of TUNEL positive cells in the si-PCSK9 group decreased greatly (Figure 3(f) and 3(g)), and flow cytometry results showed that si-PCSK9 can dramatically reduce the rate of apoptosis (Figures 3(h) and 3(i)). The above results indicated that inhibiting PCSK9 partially inhibits OGD-induced cell apoptosis.

### 3.4. Inhibiting PCSK9 Promotes the Proliferation of Neural Stem Cells

**Figure 4:**
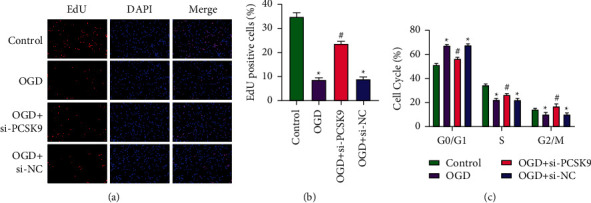
Inhibiting PCSK9 promotes the proliferation of neural stem cells. (a, b) EdU staining detected the proliferation of neural stem cells. (c) Flow cytometry detected the cell cycle of neural stem cells. ^*∗*^*vs.* control group <0.05; # *vs.* OGD + si-NC group <0.05.

EdU staining test results showed that compared with the control group, cell proliferation in the OGD group was dramatically inhibited. Compared with the OGD + si-NC group, si-PCSK9 alleviated the cell proliferation inhibition caused by OGD (Figures 4(a) and 4(b)). In the cell cycle analysis, compared with the control group, the number of cells in the OGD group was dramatically upregulated in the G0/G1 phase and dramatically downregulated in the S phase. Compared with the OGD + si-NC group, the number of cells in the OGD + si-PCSK9 group was dramatically downregulated in G0/G1 phase and dramatically upregulated in S phase (Figure 4(c)). All the results showed that inhibiting PCSK9 can promote the proliferation of neural stem cells.

## 4. Discussion

**Figure 5:**
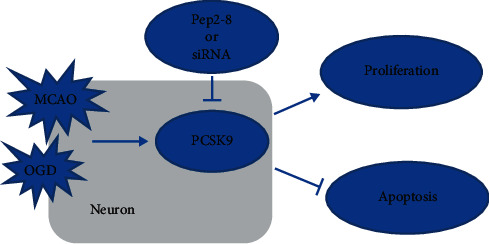
Schematic diagram of the protective mechanism of PCSK9 inhibitor on rats with middle cerebral artery occlusion-induced cerebral ischemic infarction.

In this study, we first used the rat MCAO model to find that the expression of PCSK9 in brain tissue was increased. In in vivo experiments, we found PCSK9i can improve neurobehavioral functions and reduce apoptosis and infarct volume. At the same time, neural stem cells were used to establish an OGD model, and si-PCSK9 was used to inhibit the expression of PCSK9 to clarify the role of PCSK9 in the OGD model in vitro. Our results showed that after OGD treatment, the expression of PCSK9 was dramatically increased, and si-PCSK9 inhibited OGD-induced apoptosis and promoted proliferation (Figure 5).

The mechanism of cerebral ischemia/reperfusion injury is very complicated, including inflammation, apoptosis, and mitochondrial damage. PCSK9 is a serine protease related to the regulation of blood lipids discovered in recent years [12]. It is mainly produced by the liver and then released into the blood to regulate LDL in the blood. However, recent studies have found that PCSK9 is also expressed in the cerebral cortex and hippocampus of mice. After making a mouse ischemia/reperfusion model, Rousselet et al. [13] detected that PCSK9 was dramatically upregulated. In addition, Tang et al. [14] found that PCSK9 can downregulate LDLR levels after an ischemic stroke in mice, leading to hyperlipidemia. Chiang et al. [15] also found that PCSK9 was upregulated after inducing neuronal apoptosis. The above series of experiments show that PCSK9 expression will increase after the onset of ischemic stroke, and some literatures pointed out that the role of PCSK9 in the brain and the liver may be different. In addition to participating in the regulation of blood lipids, it also has a wider range of biological effects. It is also involved in insulin resistance, inflammation, cell development, and apoptosis.

Previous studies have found that the MAPK pathway is involved in mediating PCSK9-mediated cell apoptosis [16, 17]. Through the action of shRNA, a study found that PCSK9 dramatically inhibited the phosphorylation of p-p38MAPK and JNK, [18] thereby affecting cell apoptosis. Therefore, we can guess that PCSK9 can directly induce p-p38MAPK upregulation through the MAPK signaling pathway, thereby mediating neuronal apoptosis. Our study found that si-PCSK9 inhibits OGD-induced cell cycle arrest in G1 phase, indicating that knockdown of PCSK9 may restore the normal progression of the cell cycle through its function as a spindle checkpoint in the cell cycle. However, there are some limitations in the present study. First, we only proved that inhibition of PCSK9 displayed a well correlation with I/R-induced neuronal apoptosis in vivo and in vitro, but the underlying mechanism concerning PCSK9 inhibition regulated neuronal apoptosis was hardly reported. Second, we just preliminarily evaluated the effect of PCSK9 inhibition on function and injured area in MCAO rat model, and more systematic analysis and detection are needed to assess the efficacy in in vivo MCAO rats. Thus, our further researches would aim to the in-depth investigation on the molecular mechanism and therapeutic evaluation of PCSK9 inhibition in ischemic stroke model in the future.

## 5. Conclusion

Inhibition of PCSK9 can improve the cerebral infarct volume in rats with cerebral ischemia/reperfusion injury, improve the dysfunction caused by ischemia, and protect the nerve, and its mechanism may be related to the inhibition of nerve cell apoptosis and promotion of its proliferation.

## Data Availability

The datasets used and analyzed during the current study are available from the corresponding author on reasonable request.
